# Human immunodeficiency virus (HIV)-negative uterine plasmablastic lymphoma on ^18^F-FDG PET/CT

**DOI:** 10.1259/bjrcr.20160124

**Published:** 2016-12-19

**Authors:** Jae Pil Hwang, Soo-Ho Jeong, Hee Kyung Kim, Jung Mi Park

**Affiliations:** ^1^Department of Nuclear Medicine, Soonchunhyang University Bucheon Hospital, Bucheon, Republic of Korea; ^2^^2^Department of Obstetrics and Gynecology, Soonchunhyang University Bucheon Hospital, Bucheon, Republic of Korea; ^3^^3^Department of Pathology, Soonchunhyang University Bucheon Hospital, Bucheon, Republic of Korea

## Abstract

Plasmablastic lymphoma is a rare and aggressive variant of diffuse large B-cell lymphoma with plasmablastic features, which commonly occurs in the oral cavity of human immunodeficiency virus-positive patients. Here, we present a case of plasmablastic lymphoma involving the uterus in a 54-year-old human immunodeficiency virus-negative female patient. The torso positron emission tomography/CT scan revealed intense ^18^F-fludeoxyglucose uptake in a bulky uterus with multiple sites of metastatic disease including peritoneal seeding.

## Clinical presentation

A 54-year-old female with a history of acute myeloid leukaemia, successfully treated with chemotherapy and haplo-related allo-peripheral blood stem cell transplantation, remained in remission for 4 years. A large pelvic mass was incidentally picked up on a routine follow-up CT scan of the abdomen and pelvis. The patient remained asymptomatic.

Laboratory findings showed an increased serum level of CA125 (89.2 U ml^−1^, normal range 0–35), lactate dehydrogenase (540 IU l^−1^, normal range 219–480) and immunoglobulin M (314 mg dL^−1^, normal range 40–230). Serological examination was all negative for HBsAg, human immunodeficiency virus (HIV), hepatitis C virus, HBsAb, HBeAb and HBcAb. Immunofixation electrophoresis examinations of serum and urine were both negative.

## Imaging findings

**Figure 1.**
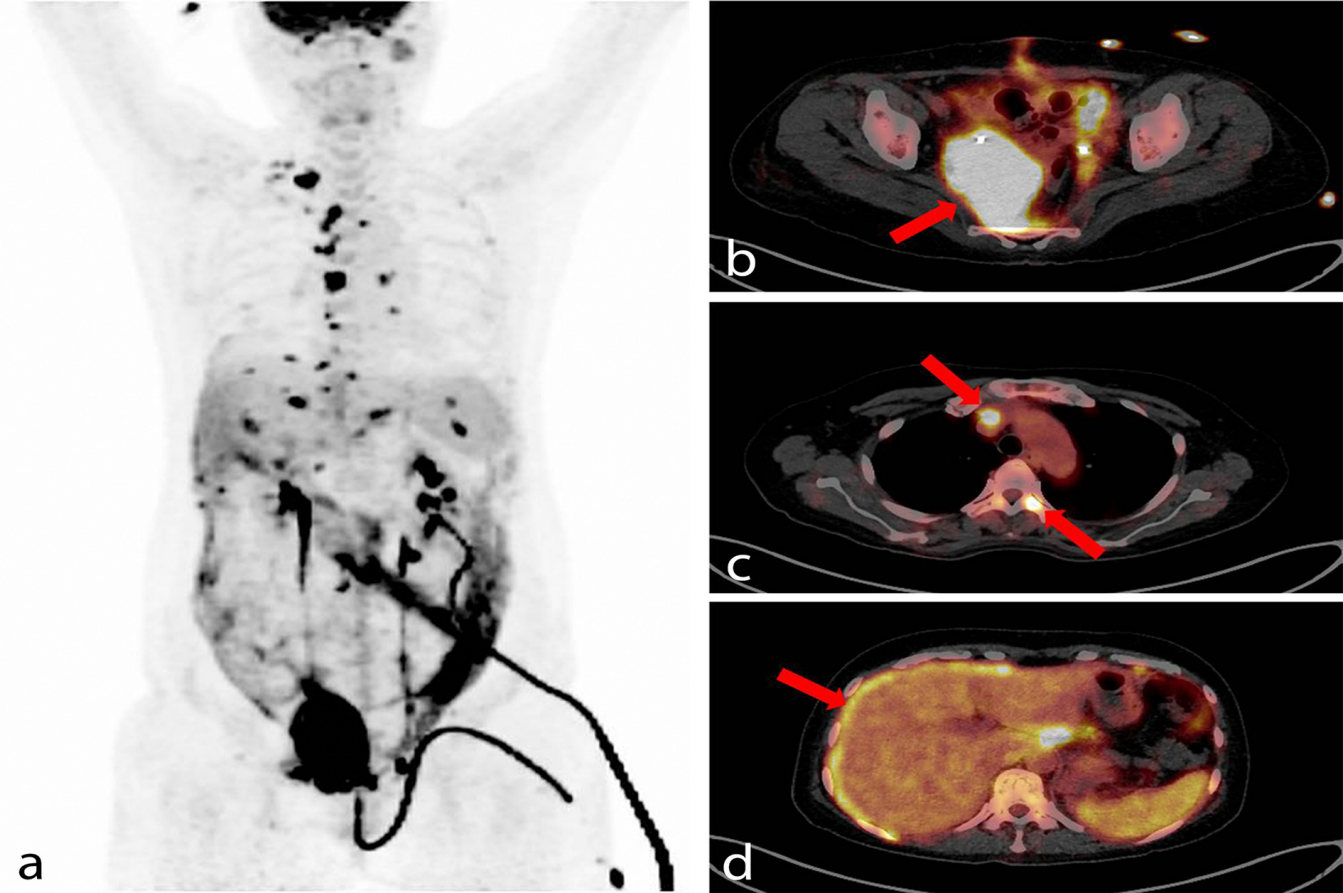
(a, b arrow) A ^18^F-fludeoxyglucose positron emission tomography/CT scan (Biograph mCT 128, Siemens, Germany) was performed for evaluation of the huge pelvic mass. The maximum intensity projection and fusion axial images show a huge intense hypermetabolic mass arising from the uterus with a maximum standardized uptake value of 37.1 and extending to the right pelvic side wall, which suggests a malignancy of uterus. (c, d arrows) Other multifocal or diffuse hypermetabolic lesions were observed in the intraperitoneal space, bones, neck and thoracic lymph nodes, suggesting multiple malignant involvement with peritoneal seeding.

^18^F-fludeoxyglucose (^18^F-FDG) positron emission tomography (PET)/CT scan showed a huge intense hypermetabolic lesion at the uterus involving the uterine cervix and right pelvic side wall. The maximum standardized uptake value (SUV_max_) of the most metabolically active portion was 37.1. ^18^F-FDG PET/CT scan revealed multifocal malignant hypermetabolic bone involvement in multiple bones (C2, T4, T6, T8, L3 spines; range of SUV_max_ 8.0–11.9), and malignant hypermetabolic lymph nodes in the neck (right cervical level V and right supraclavicular fossa), thoracic (both retrosternal, prevascular and right mediastinal region; range of SUV_max_ 6.2–18.5), presacral and intrapelvic cavity were observed. In addition, diffuse hypermetabolism in the intraperitoneal space was seen, suggesting peritoneal seeding (Figure 1).

## Treatment/outcome

**Figure 2.**
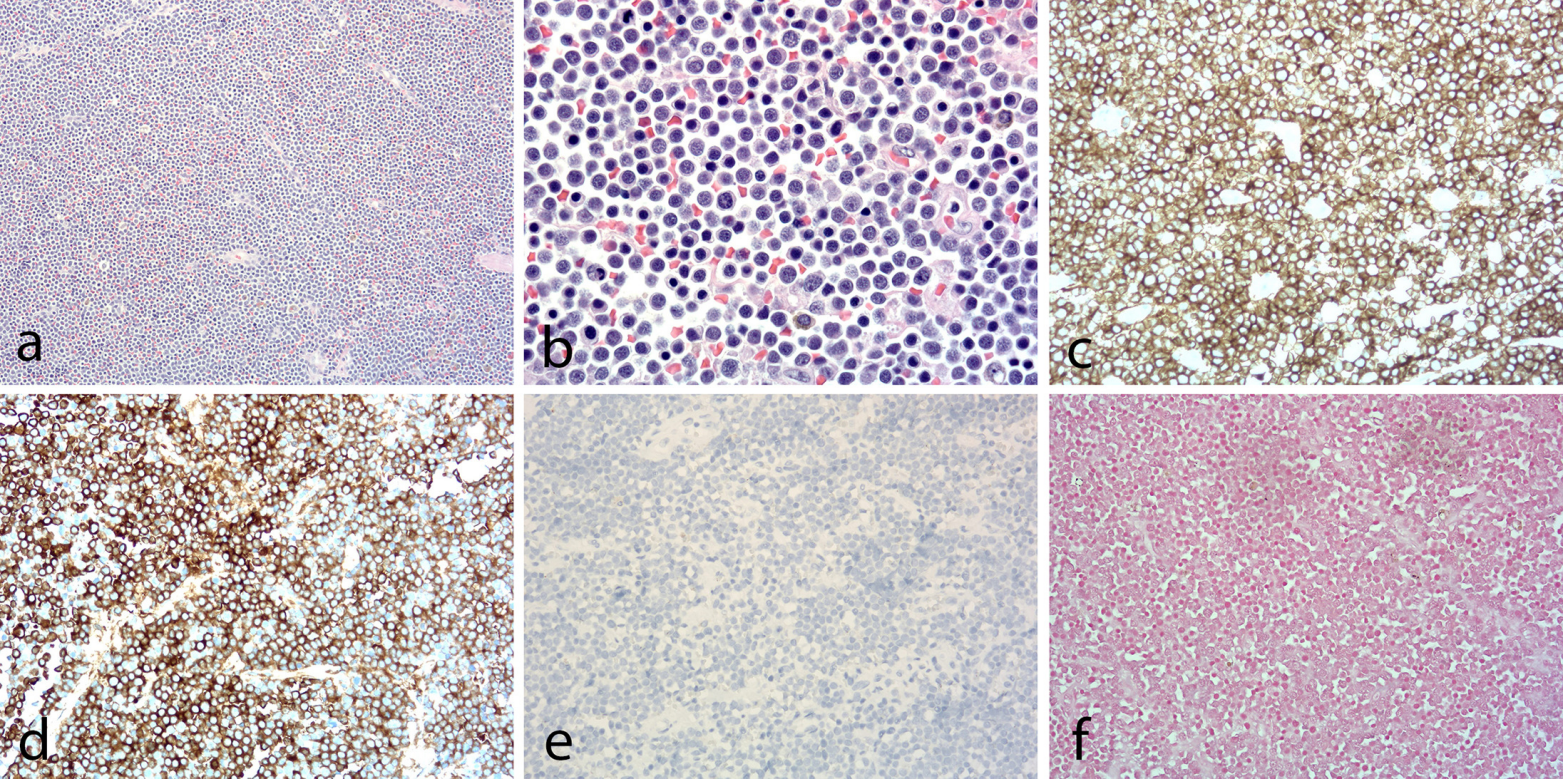
(a) Histopathology of the retroperitoneal mass revealed diffuse proliferation of monomorphic large atypical lymphoid cells (haematoxylin and eosin, ×100). (b) The tumour cells are large with eccentric nuclei, prominent nucleoli and abundant amphophilic cytoplasm. The cells in some areas resemble plasma cells (haematoxylin and eosin, ×400). (c) The tumour cells are strongly positive for the plasma cell marker CD138 (×200). (d) The tumour cells show lambda light chain restriction (×200). (e) The tumour cells are negative for CD20 (×200). (f) EBER in situ hybridisation reveals negative findings (×200). EBER, Epstein–Barr virus-encoded small RNAs.

A total abdominal hysterectomy with bilateral salpingo-oophorectomy and retroperitoneal mass removal was performed because a malignant tumour was suggested on clinical (elevated serum CA125, immunoglobulin M and lactate dehydrogenase level) and radiological (huge pelvic mass) findings. Histological findings of the retroperitoneal mass demonstrated diffuse sheets of medium to large atypical lymphoid cells (Figure 2a). The tumour cells were large with eccentric nuclei, coarse chromatin, prominent nucleoli and abundant cytoplasm, suggestive of plasmablastic differentiation. The cells in some areas resembled plasma cells (Figure 2b). Mitotic figures were frequent. On immunohistochemical staining, the tumour cells were strongly positive for CD138 (Figure 2c) and interferon regulatory factor 4/multiple myeloma 1, showed lambda light chain restriction (Figure 2d) and had more than 70% Ki-67 proliferation index, but were negative for CD3, CD20 (Figure 2e) and CD56. The Epstein–Barr virus-encoded small RNAs *in situ* hybridisation revealed negative findings (Figure 2f). This immunohistochemcial profile was consistent with plasmablastic lymphoma. The tumour extended to the cervix, endomyometrium and both adnexa. The patient was administered EPOCH chemotherapy (etoposide, vincristine, doxorubicin, cyclophosphamide and prednisone).

After four cycles of EPOCH chemotherapy, the patient underwent follow-up ^18^F-FDG PET/CT scan for treatment response assessment. Her follow-up ^18^F-FDG PET/CT showed complete remission for the previously noted hypermetabolic malignant lesions of thoracoabdominal lymph nodes and peritoneal seeding.

## Discussion

Plasmablastic lymphoma (PBL) is a very rare tumour occurring predominantly in the oral cavity in immunocompromised or HIV-positive groups.^1^

PBL should be differentiated from other malignant tumours such as gastrointestinal stromal tumours, poorly differentiated carcinomas, multiple myeloma, diffuse large B-cell lymphoma, anaplastic large cell lymphoma, Burkitt’s lymphoma and haematological malignancies such as leukaemia.^2^

A few cases of PBL using ^18^F-FDG PET/CT scan have been reported in the literature.^3–7^ To the best of our knowledge, this is the first case to report PBL of uterus using ^18^F-FDG PET/CT scan.

A ^18^F-FDG PET/CT scan was useful in evaluating the malignant potential of the uterine tumour and detection of distant metastasis for operability. Although rare, uterine PBL should be considered one of the differential diagnosis of uterine lesions detected by ^18^F-FDG PET/CT scan.

## Learning points

1. ^18^F-FDG PET/CT scan can prove the high glucose metabolism of PBL, which is a very rare and aggressive type of lymphoma.
2. ^18^F-FDG PET/CT scan is useful in identifying the treatment response of lymphoma including PBL.

## Consent

Written informed consent was obtained from the patient(s) for publication of this case report, including accompanying images.
